# Reduced Cytokine Tumour Necrosis Factor by Pharmacological Intervention in a Preclinical Study

**DOI:** 10.3390/biom12070877

**Published:** 2022-06-23

**Authors:** Armin Mooranian, Jacqueline Chester, Edan Johnston, Corina Mihaela Ionescu, Daniel Walker, Melissa Jones, Susbin Raj Wagle, Bozica Kovacevic, Thomas Foster, Momir Mikov, Hani Al-Salami

**Affiliations:** 1 The Biotechnology and Drug Development Research Laboratory, Curtin Medical School & Curtin Health Innovation Research Institute, Curtin University, Bentley, Perth, WA 6102, Australia; a.mooranian@curtin.edu.au (A.M.); j.chester@student.curtin.edu.au (J.C.); edan.johnston@student.curtin.edu.au (E.J.); c.ionescu@postgrad.curtin.edu.au (C.M.I.); danieljcswalker@gmail.com (D.W.); melissa.a.jones@postgrad.curtin.edu.au (M.J.); susbinraj.wagle@postgrad.curtin.edu.au (S.R.W.); bozica.kovacevic@postgrad.curtin.edu.au (B.K.); thomas.p.foster@postgrad.curtin.edu.au (T.F.); 2 Hearing Therapeutics Department, Ear Science Institute Australia, Queen Elizabeth II Medical Centre, Nedlands, Perth, WA 6009, Australia; 3 Department of Pharmacology, Toxicology and Clinical Pharmacology, Faculty of Medicine, University of Novi Sad, Hajduk Veljkova 3, 21101 Novi Sad, Serbia; momir.mikov@mf.uns.ac.rs

**Keywords:** diabetes mellitus, bile acids, inflammation, cytokines, interleukins

## Abstract

Recent preclinical studies in our laboratory have shown that the bile acid profile is altered during diabetes development and such alteration has been linked to the diabetes-associated inflammatory profile. Hence, this study aimed to investigate if the first-line antidiabetic drug metformin will alter the bile acid profile and diabetes-associated inflammation in a murine model of pre-type 2 diabetes. C57 mice were randomly allocated into three equal groups of eight. Group One was given a low-fat diet (LFD), Group Two was given a high-fat diet (HFD), and Group Three was given an HFD and, upon prediabetes confirmation, daily oral metformin for one month. Blood glucose, glycated haemoglobin, drug concentrations in tissues and faeces, and the inflammatory and bile acid profiles were measured. Metformin showed wide tissue distribution and was also present in faeces. The bile acid profile showed significant alteration due to prediabetes, and although metformin did not completely normalize it, it did exert significant effects on both the bile acid and the inflammatory profiles, suggesting a direct and, to some extent, positive impact, particularly on the diabetes-associated inflammatory profile.

## 1. Introduction

Type 1 diabetes (T1D) and type 2 diabetes (T2D) are characterized by the loss of control over glucose levels in the blood and the inability of tissues to regulate the amount of glucose uptake. Both diseases are encompassed by metabolic dysregulation with numerous and sometimes severe secondary effects, resulting in complications and tissue damage. Inflammation has been shown as a contributing factor to diabetes-related damage, in addition to being a risk factor for the development of diabetes itself. Inflammation induces the activation and recruitment of proapoptotic and pro-inflammatory mediators, such as TNF-α and IFN-γ. Accompanying this, oxidative stress, induced by chronic hyperglycaemia, progressively increases systemic metabolic dysregulation. The pancreatic β-cells are at exceptional risk, as they lack an adaptive immunity and antioxidant response sufficient enough to provide protection from the deleterious effects of long-term free radical exposure. The long-term outcomes of this may include nephropathy, neuropathy, retinopathy, vascular degeneration, endothelial dysfunction, and increased risk for numerous other oxidative stress-related pathologies, such as Alzheimer’s [1,2,3,4]. Such risks in T1D and T2D have been linked to the modulation of the bile acid profile.

Bile acids are endogenous cholesterol-based surfactants. The synthesis of the primary bile acids, chenodeoxycholic acid (CDCA) and cholic acid (CA), occurs within hepatocytes from precursor cholesterol in both humans and mice, directly affecting the cholesterol levels in the serum [5]. Primary bile acids are conjugated with either glycine or taurine in both humans and rodents prior to post-prandial duodenal release. Historically, bile acids have been characterized for their emulsifying effect on dietary lipids. However contemporarily, bile acids are recognized for having additional functionalities as pleiotropic messenger metabolites, regulating a diverse range of inflammatory and metabolic pathways [1]. It is understood that bile acids act as nutrient sensors, influencing the metabolic regulation of glucose, lipids, and energy release through the activation of the membrane Takeda G protein-coupled receptor 5 and the Farnesnoid X receptor (FXR). The activation of the former is associated with insulin increase and the latter is associated with reduced inflammation in diabetes. The dysregulation of bile acids within the body is associated with inflammation, obesity, diabetes, and several other metabolic diseases [6,7,8,9,10]. Furthermore, bile acid sequestrants have been found to have glucose-lowering effects in individuals with T2D [11].

Numerous studies by our laboratory have inferred a direct correlation between the variations in the bile acid pool and its distribution in T1D and T2D [4,12,13,14]. Expanding upon this, one of our recent preclinical studies explored the influence of T1D and T2D on the inflammatory profile and bile acid profile in balb/c murine models. Three groups of mice models were tested: T1D, T2D, and healthy. The concentrations of CDCA, lithocholic acid (LCA), and ursodeoxycholic acid (UDCA) were analysed in samples of blood, tissue, urine, and faeces. The results illustrated key differences in the T1D and T2D bile acid profiles when compared to the healthy murine model. Firstly, the concentrations of CDCA in T1D and T2D were significantly reduced in the intestine, plasma, and brain when compared to those of healthy mice. Secondly, the LCA concentrations within the brain and pancreas samples were elevated in the T1D and T2D models. Finally, UDCA was elevated in the T1D and T2D models, in contrast to the undetectable limits within the healthy model. The ratios of the bile acids with anti-inflammatory properties (CDCA and UDCA) were compared to pro-inflammatory LCA. Overall, there were alterations in the inflammatory profiles of the T1D and T2D murine models compared to the healthy model, in favour of reduced anti-inflammatory bile acids and increased pro-inflammatory bile acids.

Bile acids have been shown to alter drug permeability through several mechanisms. Semisynthetic bile acid 12-monoketocholic acid (MKC) has affected gliclazide permeability across the ileum in rats via selective inhibition of the multiple drug-resistant protein 3 (Mrp3) transporter. Mucosal to serosal transport was inhibited by the ileal addition of MKC in the healthy models, but the diabetic model showed no evidence of gliclazide transport at all. What this infers is that bile acids affect the permeability of transporters in the ileum, but diabetes eliminates the active control over drug transport either by way of suppression or ultimate impairment [1]. The gut biome and its composition have also been of increasing relevance with respect to metabolic disorders, bile acid distribution, and drug pharmacokinetics. Individual gut biomes vary depending on age, gender, and lifestyle, but commonalities and relationships are emerging between bile acid metabolism and metabolic homeostasis [14,15,16,17,18,19]. Fittingly, this study explored the associations among bile acid distribution in the ileal tissue, faeces, plasma, kidney, and liver, inflammation biomarker concentrations, metformin distribution, and trends connecting these in a model of pre-type 2 diabetes. Accordingly, this study aimed to investigate if metformin will alter the bile acid profile and diabetes-associated inflammation in a murine model of pre-type 2 diabetes.

## 2. Methodology

### 2.1. Materials

Metformin was purchased from Sigma Chemical Co., (St. Louis, MO, USA). The reagents and solvents required for all experiments were acquired from Scharlab S.L (Barcelona, Spain). The C57BL/6J male mice were sourced from the Animal Resource Centre (Perth, WA, Australia).

### 2.2. Pre-Type 2 Diabetes Induction in Mouse Models

All experiments were approved by the Animal Ethics Committee at Curtin University and performed according to the Australian Code of Practice for the care and use of animals for scientific purposes. The murine model consisted of male C57 wild-type mice at 5–6 weeks of age. *Ad libitum* food and water were provided over an acclimatisation period of one week. Environmental conditions entailed a 12-h light/dark cycle and a maintained temperature of 22 °C.

The mice were separated into three groups (*n* = 8; an HFD group (prediabetes model), LFD (control), and HFD–Metformin treated (M) group (1600 mg/kg/day)). The diet to induce prediabetes consisted of fructose, lard, and cholesterol (Specialty Feeds, WA, Perth, Australia), and there was a targeted blood glucose of <8 mmol/L. Metformin was given for a month [20,21], and samples were collected and pooled for analyses.

### 2.3. Plasma Cytokine Measurements: TNA-α, IFN-γ, IL-6, and IL-1β

**Figure 1:**
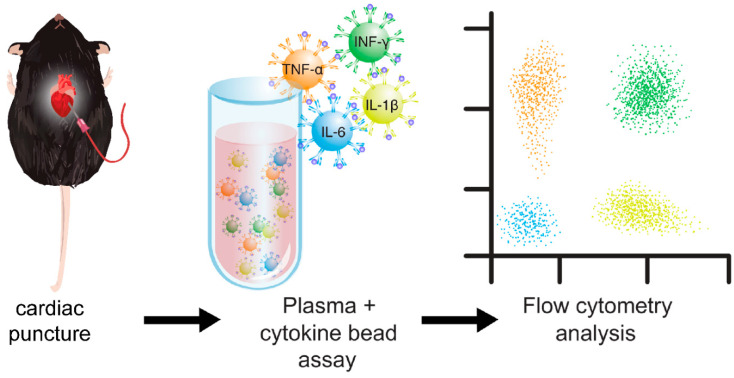
Schematic diagram of Cytokine Bead Assay (CBA) analysis used for determination of tumour necrosis factor-alpha (TNF-alpha), interferon-gamma (IFN-gamma), Interleukin-6 (IL-6), and Interleukin-1 beta (Il-1 beta).

A flow cytometry cytokine bead array kit (BD Biosciences, San Jose, CA, USA) was used to determine the plasma cytokine levels. Measurements of TNF-α, IFN-γ, Interleukin-6, and Interleukin-1β were taken using freshly thawed plasma and a BD Flex Set (BD Biosciences, Franklin Lakes, NJ, USA) following the protocols recommended by the manufacturer. The flow cytometer used was an Attune Acoustic Focusing Flow Cytometer (Life Technologies, Carlsbad, California, USA) and the data were analysed using FlowJo software version 10.8 (Ashland, OR, USA) [13,20,21,22,23]. The process is outlined in Figure 1.

### 2.4. Blood Glucose Levels (BGL) and Glycosylated Haemoglobin (HbA1_C_) Measurement

Blood samples were retrieved via the tail veins of the mice to determine the non-fasting blood glucose levels using an AccuCheck glucometer (Roche Laboratories, Gartenstrasse, Basel, Switzerland). A DCA Vantage Analyzer was used with sample DCA HbA1c reagent cartilages (Siemens Healthcare Diagnostics, Liberty Boulevard Malvern, PA, USA) [21,23,24,25,26,27]. Blood was collected via cardiac puncture for further analyses.

### 2.5. Metformin Levels

Metformin concentrations were determined in the plasma, tissues, and faeces using high-pressure liquid chromatography (HPLC). The plasma concentrations of metformin required a 20 µL plasma extraction. This was transferred with 40 µL of the mobile phase into a 1.5 mL Eppendorf tube and vortexed for 30 s. Subsequent centrifugation for 15 min at 1500 RPM was conducted, and 2 mL of the final supernatant was extracted for transfer into autosampler vials for analysis. Samples of 2 mg of both faeces and tissue from the remaining organs were collected and then added to HPLC water (2 mL). Vortexing and sonication for 30 s homogenised and extracted the drug contents from the faeces and tissue samples. A mobile phase (4 mL) was added to the samples for centrifugation for 15 min at 15,000 RPM. A 2 mL sample of the supernatant was collected and added to autosampler vials for analysis. All analyses of the samples were conducted in triplicate (*n* = 3). HPLC analysis was conducted as previously described using a Shimadzu Prominence HPLC machine (Shimadzu Corp., Kyoto, Japan), in addition to a liquid chromatographer (LC-20AT), a Shimadzu degasser (DGU-20A5), autosampler (SIL-20A), UV/Vis detector (SPD-20A), and a C-18 Phenomenex Luna column (4.6 mm × 150 mm, internal diameter of 5 µm) [26]

### 2.6. Measurement of Bile acid Profiles

A previously established liquid chromatography–mass spectroscopy (LCMS) method was used [22,23,27]. The concentrations of LCA, UDCA, and CDCA were measured in the plasma, ileum, liver, kidney, and faeces. The apparatus used for LCMS analysis consisted of a 5 µm particle size Phenomenex C-18 column (10 cm length, 2 mm internal diameter, Phenomenex Corporation, Torrance, CA, USA), with a Shimadzu LC-20 AD Prominence Liquid Chromatograph, DGU-20 A3 Shimadzu Prominence Degasser, and a Shimadzu SIL-20 AC HT Prominence Autosampler.

### 2.7. Statistical Analysis

GraphPad^®^ Prism (v.9.0.2, San Diego, CA, USA) was utilised for *p* value analyses. A *p* < 0.05 was considered significant. The values are presented as the means ± standard error of the mean. The analysis was performed using non-parametric or one-way ANOVA, with a post hoc, as appropriate.

## 3. Results

### 3.1. Inflammatory Markers and Diabetes Biomarker Levels

**Figure 2:**
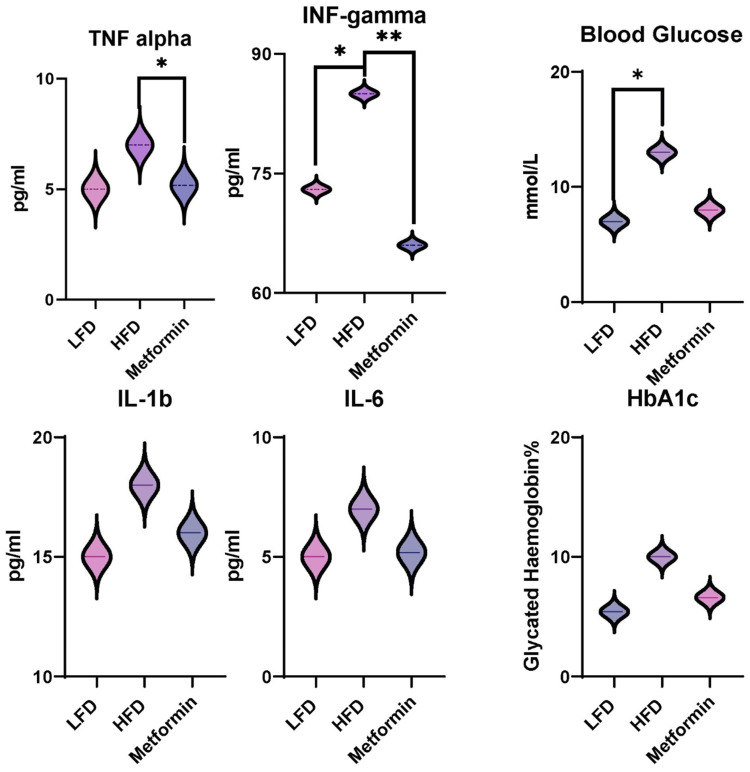
Inflammatory profile of plasma and blood glucose levels and glycated haemoglobin (**2**); *n* = 8, mean. HFD = high-fat diet, LFD = low-fat diet, M = metformin-treated, TNF-alpha = tumour necrosis factor-alpha, IFN-gamma = interferon-gamma, IL-6 = Interleukin—6, IL-1 beta = interleukin-1 beta. The data are presented as mean ± SEM (*n* = 6). * *p* < 0.05, ** *p* < 0.01.

Figure 2 outlines the recorded levels for plasma cytokines, including TNF-α, IFN-γ, IL-6, and IL-1β in all groups (Figure 2), as well as blood glucose and glycated haemoglobin (Figure 2). When comparing the inflammatory profiles of the LFD and HFD groups, it can be seen that IFN-γ was the only pro-inflammatory cytokine that was significantly different (*p* < 0.05) between the two groups (Figure 2). In the M group, TNF-α and IFN-γ were both significantly decreased in comparison to the HFD group (*p* < 0.05 and *p* > 0.01, respectively). When comparing the blood glucose levels with the HFD control group, only LFD showed a significant decrease. Metformin did not alter the blood glucose or HBA1c to any significant effect.

### 3.2. Metformin Concentration

**Figure 3:**
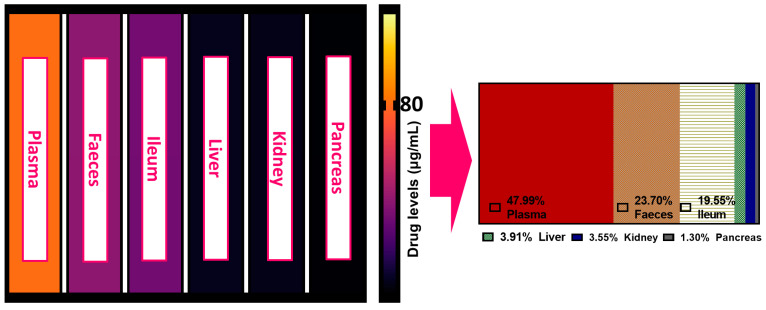
Metformin levels tested in the plasma, faeces, ileum, liver, kidney, and pancreas individually and as a ratio.

Figure 3 illustrates the accumulation of metformin in several organs and the comparative degrees of accumulation among these organs. Metformin was present in all tissues tested. The highest levels of metformin were found in the blood plasma (47.99%), followed by the faeces, which indicated a level of less than half that (23.70%) of the plasma. The concentration of metformin within the ileum was similar to that of the faeces, and finally, the concentrations in the kidney, liver, and pancreas were all low but present (<5%).

### 3.3. Bile Acid Profile

**Figure 4:**
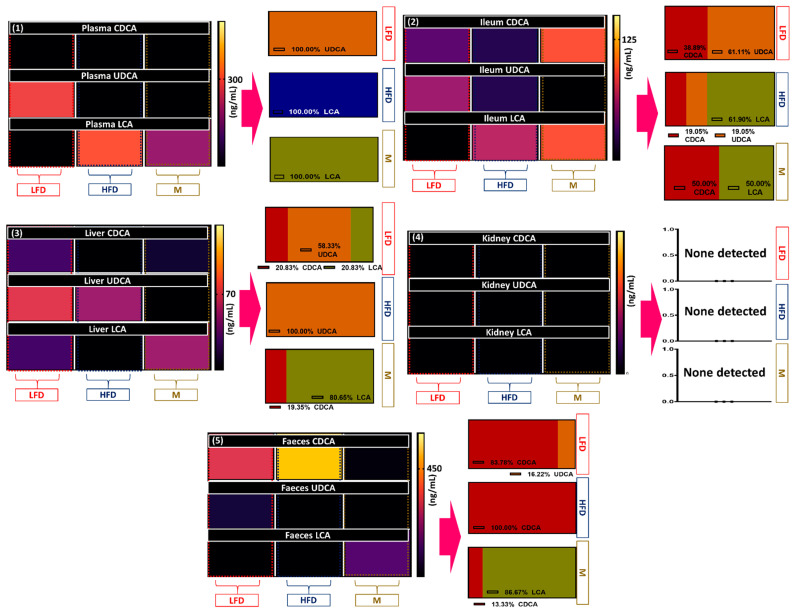
Levels of bile acids; chenodeoxycholic acid (CDCA), ursodeoxycholic acid (UDCA), and lithocholic acid (LCA) in the plasma (**1**), ileum (**2**), liver (**3**), kidney, (**4**), and faeces (**5**). HFD = high-fat diet, LFD = low-fat diet, M = metformin-treated. *n* = 8, mean values displayed.

Figure 4 shows the concentrations of CDCA, LCA, and UDCA present in the liver, pancreas, plasma, ileum, and kidney in all three experimental groups (*n* = 8). The figure shows the actual values of the drug levels within the tissues and also illustrates the ratios of the various tissues in the accumulative total concentrations of the drugs in the most comprehensive way. Within the liver of the LFD group, all three bile acids were present, with the majority consisting of UDCA (58.33%) and LCA and CDCA equally comprising the rest (20.83%). In the prediabetic model, the UDCA, LCA, and CDCA concentrations were reduced in the liver, with CDCA and LCA becoming undetectable. The plasma bile acid levels were defined, with the LFD group consisting of exclusively UDCA, whilst both the HFD and M groups contained only LCA. There was a reduction in LCA between the plasma bile acids in the M group in comparison to the HFD group, which was also demonstrated in the 6-month study. In the ileum, CDCA was present in all groups, but there was a noticeable decrease in the ratio of CDCA in the HFD group compared to the LFD group (19.05% and 38.89%, respectively). The M group demonstrated a marked increase in CDCA, accounting for 50% of its measured composition and surpassing the concentration in the LFD group. The remainder of the ileal bile acid measured in the LFD group was UDCA, which was decreased in the HFD group (accounting for 19.05%) of bile acids and undetectable in the M group. The majority of ileal HFD bile acid was LCA (61.90%), which was undetectable in the LFD group. The concentrations of LCA were further increased in the M group, accounting for 50% of the bile acids present. Finally, the composition of the faecal levels of bile acid showed similarities between the HFD and LFD groups in terms of high CDCA content, but LFD also contained UDCA (16.22%), whereas HFD consisted exclusively of the primary bile acid, CDCA. Metformin administration decreased the faecal CDCA and increased the faecal LCA, comprising 13.33% and 86.76% of the M group’s faecal bile acid composition, respectively.

## 4. Discussion

The HFD group presented higher IFN-γ (Figure 2), indicating an increased degree of inflammation within the total inflammatory profile of the HFD group. In addition, the group treated with metformin showed a significant decrease in TNF-α compared to the HFD group, showing a greater protective effect of metformin on the inflammatory profile than the inflammation associated with other groups. This inflammation due to IFN-γ appears to have been abdicated by the metformin treatment. Previous research showed significant increases in TNF-α and IL-1β in addition to IFN-γ in the HFD groups of C57BL/6J mice, but this study was conducted over a 6-month period [22]. What this may indicate is a developmental progression of the inflammatory profile expressed in prediabetes and that this may originate in IFN-γ promotion in the first month of HFD administration. Butyrate is known for its protective effect against colonic and liver inflammation via NK-FB activity inhibition. Gut microbe compositions in those with T2D showed significantly decreased butyrate-producing bacteria clusters, potentially contributing to the inflammatory profile seen in its pathogenesis [28]. For further clarification on inflammatory profile progression, future studies on the inflammatory profile at different stages with extended periods of murine HFD induction should occur.

Blood glucose and HBA1c are both used as biomarkers for the determination and diagnosis of diabetes [29]. The known hypoglycaemic effect of metformin was not of a large enough degree to result in a significant decrease in blood glucose when compared to the HFD group in this study [20,22]. This being said, the metformin group displayed lower blood glucose levels, approximating that of the LFD group far more than that of the HFD. A similar trend was observed in glycated haemoglobin levels; however, the difference between the metformin and HFD groups was less steep. It is possible that if LFD + M was also included, the effects on blood glucose and other biomarkers would have probably been substantial. Indeed, this would likely have major implications on the conclusion of the M incorporation effects and the way the control group versus the test group responded, emphasising the potentially significant impact of such an intervention.

Metformin is a molecule with low lipophilicity, indicating that its absorption is not passive in nature. Gastrointestinal absorption is considered the rate-limiting step in its bioavailability, as evidence suggests a lack of hepatic metabolism. The oral bioavailability of metformin is typically 50–60%, which is reflected in the results of this study. The faecal concentrations of metformin have been shown to fall within 20–30% of the dosage amount, which is thought to be unabsorbed and unchanged residual metformin [30,31]. The ileum has shown an affinity for metformin accumulation and has previously been shown as a major site of absorption, which explains the moderate levels detected in this study [32,33]. The liver is the main site of metformin activity, and thus, its presence in this tissue is not unexpected [31]. Metformin concentrations in the pancreas have been scarcely studied but have previously been detected [34].

The bile acid profiles in healthy, prediabetic, and metformin-treated murine models were explored. There was a noticeable alteration in the distribution of the bile acids in all tested tissues except the kidneys, where no bile acids were detected in any group. The primary bile acid CDCA and the secondary bile acid LCA were analysed, in addition to the secondary bile acid UDCA. The ratio of CDCA to LCA highlights the distribution of the major primary and secondary bile acids within the body [35]. It is acknowledged that LCA possesses hepato-toxic and membranolytic properties, whereas UDCA and CDCA possess anti-inflammatory properties and actions [6,36,37,38]. The authors of this study assume that the effects demonstrated are clinically relevant, but it should be noted that there are variations in murine bile acid profiles compared to humans. In addition, unique murine primary bile acids are also synthesised within hepatocytes, including α-muricholic acid (α-MCA) and β-muricholic acid (β-MCA), using CDCA as its precursor and reducing CDCA ratios in contrast to those of humans [7,39].

The synthesis of the primary bile acids CA and CDCA in both humans and mice is instigated by the enzymatic action of the p450 isoenzyme, CYP7A1. Diabetes has been associated with increased total bile acids, and this has previously been attributed to hyperglycaemia-induced CYP7A1 histone acetylation [40]. The distribution of bile acids in healthy murine models supports the previous research by our laboratories exploring T1D and T2D murine bile acid profiles in comparison to healthy murine models. Contrasting the liver bile acid composition of T2D, the prediabetic model demonstrated a reduction in LCA. Similar to the variation shown in the cytokine profile in comparison to previous research, this may indicate a progression of disease-dependent changes in the bile acid pool metabolism and may be further clarified in a study of a longer duration. Alternatively, this may be indicative of increased CDCA dehydroxylation and a subsequent increase in LCA, leading to enterohepatic circulation and resulting in liver accumulation.

A previous 3-month study evaluating the bile acid composition in T2D murine models induced via HFD and alloxan injection demonstrated alterations in plasma bile acids in favour of LCA concentrations with reductions in serum CDCA and UDCA. This is supported by the results of the current study, in addition to showing these changes occurring within a period of 1 month. Changes in the plasma bile acid composition in the metformin-treated group are similar to those shown in the previously mentioned 6-month study [20].

After the conjugation of primary bile acids within the liver and storage in the gall bladder, bile acids are postprandially released into the duodenum to assist with the chemical breakdown and absorption of lipids. On the luminal border of enterocytes in the ileum, apical sodium-dependent transporters (ASBT) transporters facilitate the active transport of conjugated primary bile acids into the enterocyte. These bile acids transport out of the cell via basolateral organic solute transporters alpha and beta (OSTα and OSTβ) [7]. FXR activation and TGR5 activation is induced, in order of the affinity of the bile acids: CDCA > TCA > DCA and LCA > DCA > CDCA = CA, respectively [41]. Within the enterocytes, the activation of FXR receptors inhibits ASBT and promotes OSTα and OSTβ activity, enhancing the transcellular transport into the portal vein for mobilisation to the liver. Within the liver, hepatic FXR activation induces CYP7A1 and CYP81B inhibition, reducing bile acid synthesis and CDCA hydroxylation into CA [42]. The presence of CDCA and UDCA in the ileum is expected in the LFD group, mirroring previously demonstrated bile acid profiles of healthy murine models. Some studies have shown the presence of LCA, but this has been in low proportions compared to CDCA and UDCA [4,20]. The increase in LCA in the HFD group may be due to varying factors. LCA is synthesized by the dehydroxylation of CDCA by the enzymatic action of 7-α hydroxylase (7-ADH), predominantly actioned by the *Clostridium* genera [43,44]. Prior to transformation into a secondary bile acid, primary bile acids require deconjugation, which requires bacterial bile salt hydrolase (BSH) activity. All predominant divisions of bacteria contain BSH-capable bacteria, including Firmicutes, Actinobacteria, Bacteroidetes, and Archaea [43,44,45,46]. Increased ileal LCA and decreased ileal UDCA and CDCA in the HFD group may indicate either a reduction in the passive reabsorption of unconjugated LCA in the colon or alteration in the enzymatic transformations in the ileum. UDCA can be made via transformations of both CDCA and LCA via different enzymes expressed by the microbiome. This may indicate changes in the enzymatic activity in the gut in favour of CDCA transformation into LCA and a reduction in UDCA synthesis via both potential pathways, or alternative effects controlled by the bile acid feedback biological effects resulting from the transhepatic recirculation [47]. 

Several reviews have explored the link between diabetes and microbial dysbiosis, with studies highlighting increases in inflammation-related bacteria and their by-products [48,49,50]. Increases in LCA and a lack of UDCA in the M group ileal tissue are consistent with previous studies, and metformin has shown a proliferative effect on several BSH bacteria, which increases the primary bile acid availability for dehydroxylation [17,51]. The promotion of *Clostridium* spp. growth by metformin may explain the increase in LCA compared to that in the HFD group [52]. This may also contribute to the decreased UDCA detected in the ileum of the M group. Another explanation may be the metformin-mediated suppression of *Bacteroides fragilis* growth. UDCA conjugation into taurine-UDCA (TUDCA) and guanine-conjugated UDCA (GUDCA) has shown to be reduced in the presence of *B. Fragilis*, so its suppression encourages conjugative action [53]. The secretion of glucagon-like peptide-1 (GLP-1) has antidiabetic effects, and the bile acid receptors FXR and TGR5 have shown abilities in its modulation [54,55]. GLP-1 inhibition appears to be a direct effect of FXR activation, but indirectly, it increases GLP-1 secretion via the metabolic alteration of the bile acid pool in favour of secondary bile acids, subsequently activating the GLP-1-promoting TGR5 receptor [56,57]. Several bile acids have shown hypoglycaemic effects in pharmacological contexts in addition to providing cytoprotective effects in hyperglycaemic environments. GLP-1 modulation may be a contributing factor to this effect [58,59]. TUDCA and GUDCA are both FXR antagonists, whereas LCA is an FXR agonist and a potent activator of the TGR5 receptor [41,53]. The individual action or interaction of these bile acids with the FXR and TGR5 receptors may contribute to the hypoglycaemic effects of metformin [5,60].

The excreted bile acids in the LFD group correlate with previous studies in all respects except in LCA concentration, which was not detected [20]. A lack of LCA detected in the HFD faecal sample may infer an increase in the reabsorption of LCA into enterohepatic circulation, potentially contributing to the increased inflammatory state of the HFD model. The presence of the exclusively primary bile acids in the faeces of the HFD group may be explained by the microbial composition changes in T2D pathogenesis. Firstly, overfed mice showed a decreased habitation of BSH-active Bacteroidetes [18]. The gene expression of BSH Firmicutes, in addition to Firmicute-derived 7-ADH gene expression, was decreased in those with T2D. A result of this would be a decreased deconjugation of the primary bile acids, subsequently decreasing the dehydroxylation of the primary to secondary bile acids within the gut [28]. Comparatively, the M group demonstrated increased LCA levels at higher concentrations than those detected in the ileum. Metformin was previously shown to suppress bile acid reabsorption and to facilitate bile acid excretion, which may lead to the increased dehydroxylation of the primary bile acids into secondary bile acids within the large intestine, subsequently increasing excretion [61].

### Bile Acid Levels and Inflammatory Profile Crosstalk

The FXR receptor has multifaceted abilities in the regulation of the diabetic condition. FXR expression in diabetic Zucker rat and streptozotocin-administered aged diabetic rat hepatocytes was decreased, but this was ameliorated by insulin administration [62]. In addition to the previously mentioned effects on glucose and bile acid metabolism, FXR receptors have also shown extensive functions in inflammatory response and mediation. Insulin resistance has previously been linked to the accumulation of proteins in hepatocytes, inducing inflammation via the activation of the (NK-FB) pathways. The activation of the FXR receptor has shown anti-inflammatory effects via the inhibition of the NK-FB-associated inflammation in several locations [63,64,65,66,67]. In addition, the expressions of pro-inflammatory cytokines IL-1β, IL-6, and TNF-α are attenuated by FXR and TGR5 activation [65]. Conversely, pro-inflammatory cytokines exert inhibitory effects on FXR activation, allowing for the perseverance of NK-FB signalling and associated inflammatory damage. This may lead to compounding cyclical chronic inflammation, potentially similar to that seen in T2D [66]. These effects may be associated with the ability of metformin to decrease pro-inflammatory cytokines in prediabetic mice models. [67,68,69,70,71,72]. This study did not measure the expressions of FXR, TGR5, or NFKB proteins but the evaluation of these pathways will be a future direction of study.

## 5. Conclusions

Metformin showed dispersion in all tested tissues, in addition to exerting anti-inflammatory effects. There were marked differences between the bile acid profiles of healthy mice and those with an induced prediabetic state. These profiles were further altered by metformin. These findings suggest an interplay among prediabetes development and progression, bile acids, and the inflammatory profile in our mouse model.

## Data Availability

The data presented in this study are available upon request from the corresponding author.
